# *Simulium* larvae susceptibility to temephos and the effect of 10 weeks of treatment of the Mbende tributary in the Nkam-Wouri River drainage of Cameroon on larval density and adult fly biting rates

**DOI:** 10.1186/s13071-025-06837-5

**Published:** 2025-07-01

**Authors:** Raphael Awah Abong, Relindis Ekanya, Theobald Mue Nji, Fanny Fri Fombad, Glory N. Amambo, Innocentia Ngong, Franck Noel Nietcho, Kebede Deribe, Benjamin Biholong, Flobert Njiokou, Same Ekobo, Charles Wondji, Peter Enyong, Samuel Wanji

**Affiliations:** 1 Department of Medical Laboratory Sciences, Faculty of Health Sciences, University of Buea, P.O. Box 63, Buea, Cameroon; 2 Research Foundation in Tropical Diseases and Environment, Buea, P.O. Box 474, Buea, Cameroon; 3 Department of Sociology and Anthropology, Faculty of Social and Management Sciences, University of Buea, P.O. Box 63, Buea, Cameroon; 4 Parasites and Vector Research Unit (PAVRU), Department of Microbiology and Parasitology, University of Buea, P.O. Box 63, Buea, Cameroon; 5 Department of Microbiology and Parasitology, Faculty of Science, University of Buea, P.O. Box 63, Buea, Cameroon; 6 School of Public Health, Addis Ababa University, Addis Ababa, Ethiopia; 7 Department of Disease Control, Epidemics and Pandemics, Ministry of Public Health, National Program for Onchocerciasis Control, Yaoundé, Cameroon; 8 Parasitology and Ecology Laboratory, Department of Animal Biology and Physiology, Faculty of Science, University of Yaoundé 1, P.O. Box 812, Yaoundé, Cameroon; 9 Centre for Research in Infectious Diseases (CRID), P.O. Box 13501, Yaoundé, Cameroon

**Keywords:** *Simulium*, Larval susceptibility, Larviciding, Biting rates

## Abstract

**Background:**

Despite over 18 years of annual ivermectin mass drug administration (MDA) in Cameroon’s Nkam-Wouri River drainage, onchocerciasis transmission persists. Several reasons, including multiple breeding sites and abundant vector populations, contribute to ongoing transmission. High vector abundance also causes a biting nuisance to local populations. The change in paradigm from onchocerciasis control to elimination may not be achieved if alternative control measures are not used. There is a need to complement ivermectin MDA with other strategies. This study tested the susceptibility of *Simulium* larvae to temephos insecticide and monitored the effect of 10 weeks of ground larviciding on the larval density and black fly population.

**Methods:**

*Simulium* breeding sites along the course of three rivers within the Solle transmission zone in the Nkam-Wouri River drainage were identified. Seven temephos concentrations (0.001–0.1 mg/l) were tested on freshly collected *Simulium* larvae for susceptibility. *Simulium* biting rates were monitored using human landing catches before and during 10 weeks of ground larviciding. Fishing was used to assess the abundance and diversity of large aquatic fauna, while the presence and diversity of small invertebrate fauna were assessed during the collection of larvae, as they are usually found on the same substrates in the river. Ground larviciding was conducted using the spraying method at two dosing points.

**Results:**

Six breeding sites were identified. Larval mortality decreased with temephos concentration, with 100% mortality observed at 0.1–0.025 mg/l. The non-target fauna included various fish species, crabs, crayfish, and small invertebrates. Ground larviciding cleared larvae from identified substrates and reduced adult fly biting rates by 82.8% (from 900 flies/man/day at the beginning to 180 flies/man/day at the end), a statistically significant decrease (*χ*^2^ = 1351.5, *P* < 0.001).

**Conclusions:**

*Simulium* larvae showed susceptibility to temephos. Clearance of larvae from traps and identified natural substrates, and a significant reduction in the *Simulium* biting rates were observed.

**Graphical Abstract:**

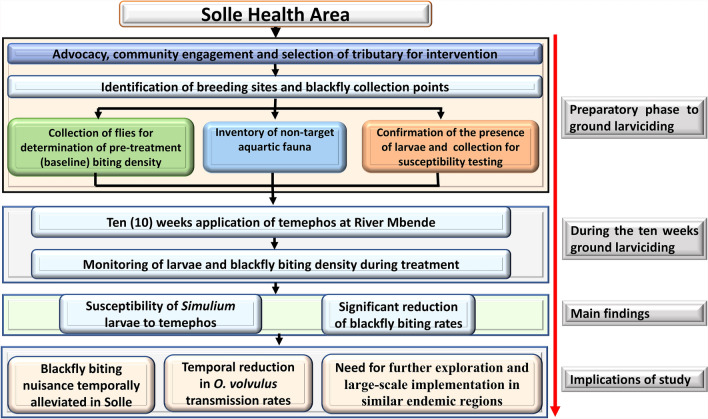

**Supplementary Information:**

The online version contains supplementary material available at 10.1186/s13071-025-06837-5.

## Background

Onchocerciasis (river blindness) is a parasitic disease caused by the filarial nematode *Onchocerca volvulus* [1]. Adult worms live in subcutaneous nodules and produce millions of microfilariae during their reproductive lifespan of 10 to 15 years [2, 3]. The microfilariae can live between 1 and 2 years in the skin, from which they are taken up by *Simulium* (black fly) vectors during a blood meal [3, 4]. Microfilariae develop into infective larvae within black flies, which infect another person during a subsequent bite, thus maintaining the transmission cycle [3].

Onchocerciasis, one of 21 priority neglected tropical diseases (NTDs) targeted for elimination by 2030 [5], is still an important public health problem in forested areas co-endemic for loiasis [6, 7]. A study revealed that the prevalence in the rain forest zone of Cameroon (South-West region) is as high as 52% [8]. The people most at risk of acquiring onchocerciasis are those who live or work near fast-flowing streams or rivers with breeding sites for *Simulium*. Studies have also reported that black fly vectors are a source of nuisance in communities located near breeding sites, notably through their painful or itchy bites, which mostly result in skin marks [9, 10]

The Onchocerciasis Control Programme (OCP) successfully eliminated onchocerciasis as a public health problem in the savanna regions of 11 West African countries [11] through vector control and ivermectin treatment before its closure in 2002 [12]. Outside OCP areas, control of onchocerciasis in Africa had been the responsibility of the African Programme for Onchocerciasis Control (APOC), where the distribution strategy for ivermectin has been community-based [13].

Despite extensive efforts by the OCP and APOC to control onchocerciasis, transmission persists in some endemic foci, particularly in forested areas of Cameroon, even after 18 years of annual community-directed treatment with ivermectin (CDTi) [6, 14–16]. Several factors contribute to this persistent transmission, including severe adverse events in *Loa loa* co-endemicity areas [4, 8], rapid skin repopulation following ivermectin treatment in microfilaridemic individuals [14], and sustained transmission indices even after annual mass drug administration (MDA) campaigns [15]. The presence of breeding sites throughout both rainy and dry seasons in some endemic foci further complicates control efforts [15]. These challenges suggest that relying on annual mass drug treatment with ivermectin alone may be insufficient for achieving onchocerciasis control, let alone elimination. Motivated by the recommendation of the World Health Organization and supporting studies [17, 18], there is growing interest in complementing CDTi with alternative control strategies, including enhanced community-directed treatment, community-directed treatment with drug combinations, test and treat (TnT), and vector control. This study focused on vector control through ground larviciding with temephos, an organophosphate larvicide that affects the nervous system of black fly larvae through inhibition of cholinesterase [19]. Temephos was selected as the first choice because it is environmentally tolerable and biodegradable, with negligible damage to the non-target aquatic fauna for all solutions with ≤ 1 m/l [20]. Ground larviciding shows promise to accelerate the elimination of onchocerciasis in forested endemic foci like that of the Yabassi health district with persistent transmission [16, 21]. This method is especially suitable for forest areas, as tree cover can interfere with aerial spraying methods used in the savanna regions. This study aimed to evaluate the efficacy of temephos in killing *Simulium* larvae and reducing adult fly biting rates in our study area, providing evidence for the potential of ground larviciding with temephos as a complementary strategy to annual ivermectin MDA in accelerating onchocerciasis elimination.

## Methods

### Study area

This study was carried out in Solle village, a first-line community in the Solle Health Area, located within the Nkam-Wouri River drainage in the Yabassi health district of the Littoral Region, at 100 km north-east of Douala, the economic capital of Cameroon. According to the 2014 census by community drug distributors (CDDs), Solle village had a population of 21,459 inhabitants. The topography is undulating, showing an alternation of valleys and plains. The altitude of the region varies from 10 to 800 m above sea level. This district is irrigated by many fast-flowing rivers, including Nkam, Dibombe, Mbende, Dimbong (Bissongo), and Mabombé (Njanga) rivers, which are favourable for black fly breeding. The vegetation is mainly dense, humid forest. At least 80% of inhabitants are involved in agriculture, making it the main economic activity.

### Community engagement and advocacy meetings

Upon obtaining the ethical clearance and administrative authorization for this study, community engagement and advocacy meetings were organized at the Solle Integrated Health Centre (IHC) with members of the research team and 22 local participants, including the representatives of the Chief of Health District and Chief of Bureau Health, Community leaders of Solle and Dimbong villages, local religious authorities, Chief of Centre for the Solle IHC, nurses, fishermen, quarter heads, CDDs, town criers, the leader of the community “vigilante group”, and the Gendarmerie brigade commander’s representative. This activity aimed at fostering greater community involvement in the design, governance, and delivery of the project. By facilitating local ownership and joint accountability, it sought to enhance programme sustainability. Additionally, it promoted collaborative partnerships, bidirectional communication, and mutual learning, ensuring that the voices and agency of local communities were meaningfully incorporated into public health practices. The objectives and procedure of the study were explained to participants, and all stakeholders consented by unanimously making promises to collaborate with and give the research team the desired assistance throughout the project’s implementation.

### Study design

**Fig. 1:**
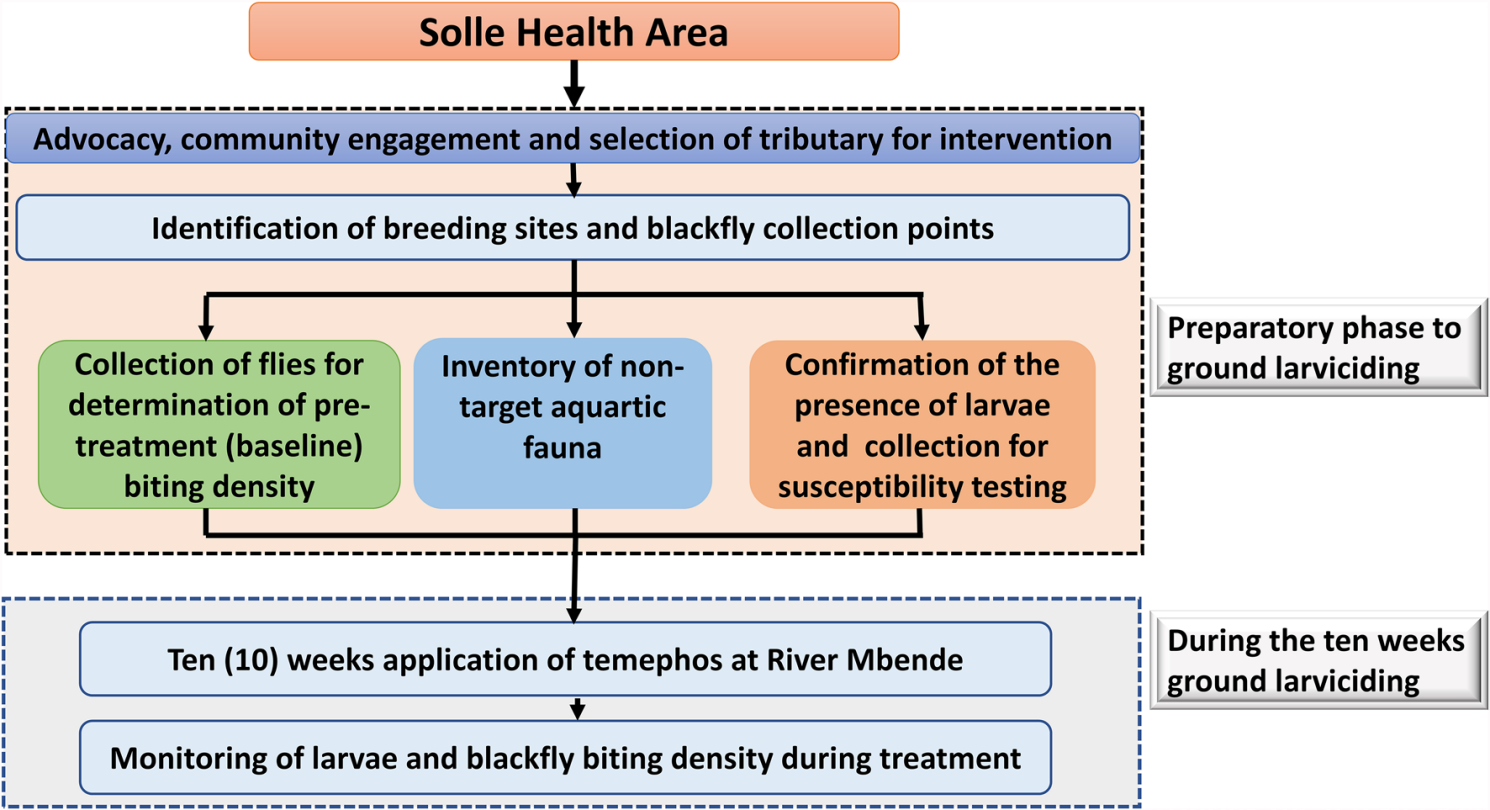
Study design

This was a before–after study, designed to establish a proof of principle for the implementation of ground larviciding in a forested endemic zone. The study period spanned from May 2021 to January 2022, cutting across the rainy/wet and dry seasons in Cameroon. Baseline entomology indices were collected during the wet season. Larva susceptibility tests and 10 weeks of ground larviciding were conducted during the dry season when the water volume was relatively stable. The effect of 10 weeks of ground larviciding was observed with respect to the larval density and *Simulium* fly biting rates during the treatment period. Figure 1 summarizes the design used during the study from breeding site identification to monitoring *Simulium* larvae and adult biting density during ground larviciding.

### Breeding site identification within the Solle Health Area

*Simulium* larvae breeding sites were identified mainly by prospection along all three rivers within the Solle transmission zone. Each river was checked for the presence of rapids (areas with high water speed, which have increased oxygen flow) and trailing vegetation (substrates). The presence of one or all of these indicates that the site is a potential breeding site. During this time, coordinates were taken using the Global Positioning System (GPS; Garmin GPSMAP 65s—portable GPS multi-band/multi-GNSS [Global Navigation Satellite System]).

### Study site in Solle

Considering that the Mbende River (a tributary of Mabombé) had characteristic features of *Simulium* breeding, flowing across Solle village and being more accessible than the other rivers in the area (the transmission zone), it was selected as the study river, while the Bissongo/Dimbong River was chosen for comparison of entomological indicator (biting rates) before ground larviciding.

### Selection of temephos dosing points in the Mbende River

A survey of the river system 10 km upstream and 3 km downstream from the bridge in Solle village was performed to observe the presence of trailing vegetation and rapids. Also, the trailing vegetation and other substrates were checked for the presence of *Simulium* larvae. Temephos dosing/treatment points were chosen about 100–200 m upstream from the identified breeding sites, depending on accessibility and the possibility of navigating across the river. Coordinates of treatment points were taken using a GPS device.

### Sensitivity testing

Traps of palm branches were set by immersing them in the river at a point of rapid water flow (potential breeding sites), at least 2 weeks before the day of testing (Fig. S1). *Simulium* larvae were collected from the set traps and natural substrates (fixed submerged vegetation, water plants, trailing roots and branches, fallen leaves, stones, broken tree branches, and inclined rock surfaces) for sensitivity testing.

*Simulium* larvae have been reported to survive out of their breeding site for more than 4 h (3–6 h) when the temperature is maintained in the range of 21–23 °C, but exposure to larvicide for 3 h gives the best results [22]. The entire substrates containing larvae were placed into a bowl containing river water and transported to the testing site within 30 min of collection in a cooler without agitation. From the bowl, a hand lens was used to identify the fourth (presence of very small and only just differentiated leg and wing buds) and fifth (presence of light-coloured or whitish gill spots and well-separated leg and wing buds) larval instars [23] that were used for susceptibility testing. Seven concentrations of temephos/Abate from 0.001 to 0.1 mg/l (0.1, 0.05, 0.025, 0.01, 0.005, 0.0025, and 0.001 mg/l) were tested alongside the control groups (larvae in river water with no insecticide) under the same conditions. Just before pouring, each container of Abate was mixed by shaking vigorously to ensure an even distribution of the solution and to introduce air bubbles. Forty (40) larvae were counted, picked with a soft forceps, and put into 250 ml of temephos (for the test) or river water (for the controls) in a 500-ml bowl. The larvae were placed in the bowls containing temephos as soon as they were brought from the river (≤ 30 min from the time of collection) and incubated under a controlled temperature of 23–25 °C for 3 h (Fig. S2.). The larvae were not fed any artificial diet, and for each temephos concentration, three replicates were made. Following the incubation period, the number of larvae living in each bowl was counted as described by Kalinga and colleagues [24]. A criterion for larval mortality adopted by the World Health Organization (WHO) is “absence of reaction to a pinprick after the incubation period” [25].

### Fly collection at baseline

Two fly collection points were established, each staffed by two trained dark-skinned collectors who worked alternating shifts. One team was at the bank of the Mbende River (study river) where treatment (ground larviciding with temephos) was conducted, and the other along the bank of the Dimbong River. Preliminary fly collection was carried out in June and July 2021. Flies were caught on an hourly basis from 7 am to 5 pm for four consecutive days during the June field visit and for eight consecutive days during the July field visit. The monthly biting rates (MBR) for June and July were determined as described in earlier studies [26]. The hourly and daily biting rates were determined, and the flies were stored in 80% alcohol for future use.

### Picture of vertebrate non-target fauna at Mbende River

Four local fishermen were briefed on the expectations of the study. The information sheet was explained to them, and they signed the consent form to voluntarily participate in the investigations. They were asked to provide a list of aquatic vertebrates that they commonly saw and caught in the Mbende River. Also, they were given the task of fishing and presenting everything that was trapped in their nets to the research team for two consecutive days to assess the abundance and diversity.

### Ground larviciding

Ground larviciding involved prospection and identification of treatment points, calculation of river discharge rates, and application of temephos in the river.

#### Prospection of treatment points

Prospection was done by moving along the river course to identify breeding sites based on the rapids. Treatment points were marked at river points above many rapids, which were accessible and where larvicide could be easily applied across the river during treatment.

#### Calculation of river discharge

The river discharge rate was calculated at two points: 20 m above the bridge in Solle village and 5 km upstream from the bridge. The calculations were based on the river width, the average depth, and mean speed measurements at both treatment/dosing points as described below.

The width (W) was determined by measuring the distance across the river using a graduated rope.

The mean speed (V_m_) was calculated using the floating technique, where a visible piece of polystyrene material was placed on the river surface, and the time that it took to move from point A to point B was recorded. This was repeated five times, and the surface speed (V_s_) was computed. The length/distance between points A and B (20 m) was measured by two individuals along the banks of the river.

Given that the speed of the water at the surface (V_s_) is usually faster than the speed at the bottom, the mean speed (V_m_) was calculated by multiplying V_s_ by 0.8, where 0.8 is the correction coefficient.

The depth was measured by inserting a graduated stick into the river at different points (every 2 m) along its width, and the average of the readings was calculated to obtain the average depth (A_d_).

Therefore, the river discharge (Q) was calculated using the formula Q = A_d_ × V_m_ × W, and the result is expressed in cubic metres per second (m^3^/s).

#### Preparation of the quantity of temephos sprayed

The amount of temephos insecticide sprayed depended on the dose of insecticide and the river discharge/flow rate [27] The formula is: quantity = dose (l/m^3^/s) × discharge (m^3^/s). Using 0.3 l of Abate/m^3^, the volume of Abate in 10 l solution was computed by multiplying this factor (0.3) by the computed discharge rate (Q).

#### Treatment (application of temephos)

Two larvicide applicators were recruited and trained to treat the river using the spraying method. The treatment protocol involved measuring the required quantity of temephos (0.3 × discharge rate), mixing it with river water to obtain a total volume of 10 l in a 15-l sprayer, and applying it into the river within 7–10 min. Due to the toxicity or corrosiveness of undiluted temephos, proper personal protective equipment was worn during all applications. Temephos application was done weekly (every seventh day) for 10 weeks, from 12 November 2021 to 5 January 2022. This 7-day interval was chosen because black flies complete their larval development in 8–10 days when temperatures are below 35 °C [27].

### Assessing the effect of ground larviciding

Traps (palm branches) and other substrates identified with larvae were checked/observed every week (on the eve of treatment day) throughout the treatment period for the presence or absence of larvae. The effectiveness of ground larviciding was evaluated through two main methods: larval monitoring on the traps and/or natural substrates, and adult *Simulium* biting rates at the riverbank during the 10-week treatment. Black flies were collected at different time points (from the eve of the first day of temephos larvicide application to the eve of the 10th treatment day), and biting rates/densities were calculated.

### Data analysis

Fly collection data were entered in a template created in Microsoft Excel 2013 for analysis. The different entomological indicators generated were biting densities, DBR, and MBR. Biting density was the number of flies collected at different hours plotted against the hours collected. The adjusted mortality of *Simulium* larvae for each concentration was calculated utilizing the control mortality frequency that ran in parallel with the test. If the control mortality was 5–20%, then Abbot’s formula [26] was used to correct the mortality and ensure that other factors were not contributing to the mortality of the larvae, as follows:

$$\text{Corrected test mortality }= (\text{\%test mortality }-\text{ \%control mortality})/(100 -\text{ \%contol mortality})\text{ x }100$$

No adjustment was made if control mortality was below 5%, and results were discarded for any testing with control mortality above 20%. The temephos dosage-dependent mortality curve was constructed using GraphPad Prism 7.0 (GraphPad Software, San Diego, CA, USA). A Chi-square test was performed using R software (version 4.3.3) to compare biting densities at different time points during the 10-week treatment period.

## Results

### *Simulium* breeding sites in the Solle Health Area

**Fig. 2:**
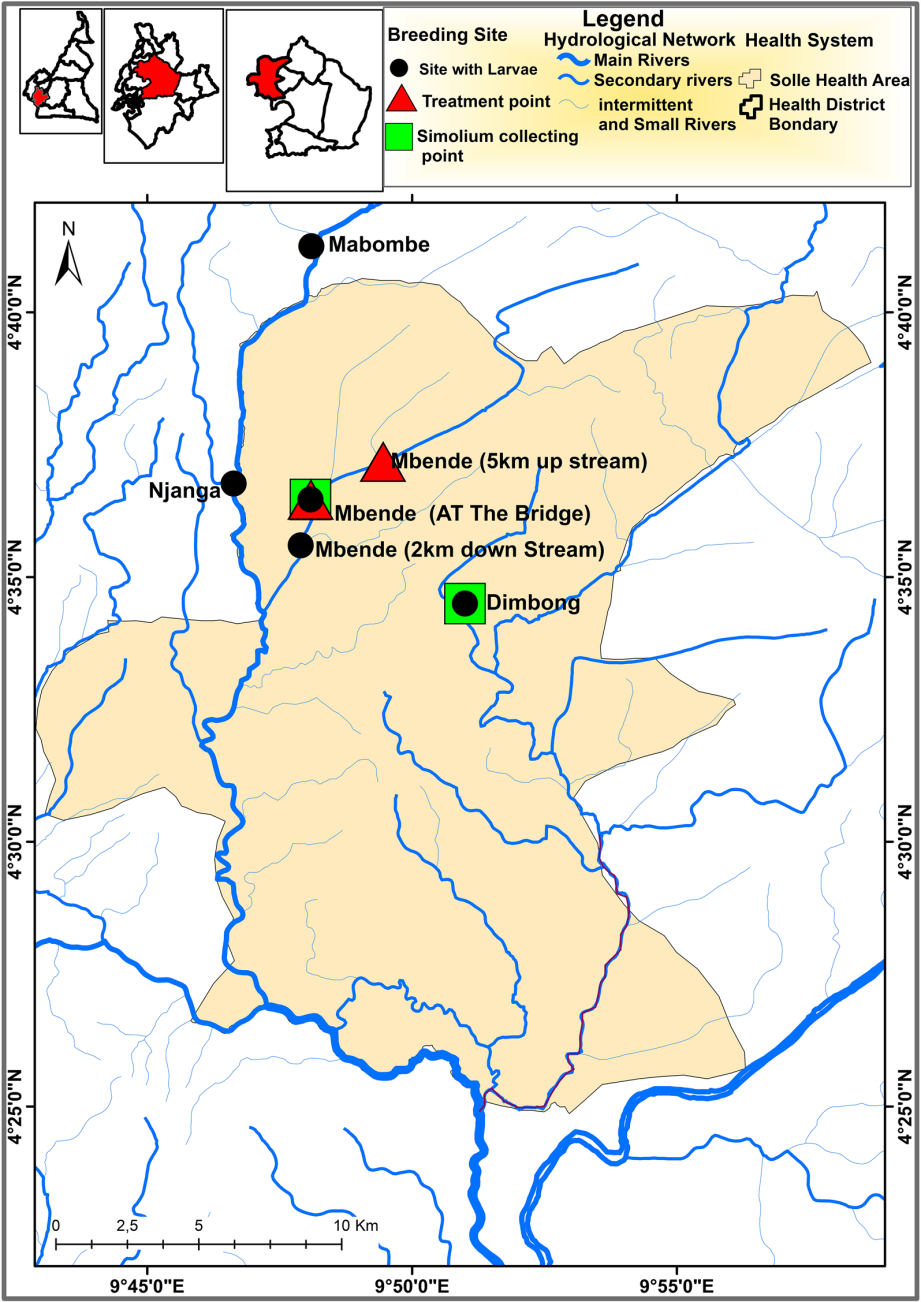
Map showing identified *Simulium* breeding sites and temephos treatment/application points in the Solle Health Area

Six breeding sites for *Simulium* were identified in the Solle Health Area (HA), and five of them were productive for larvae, as shown in Fig. 2. We did not collect larvae at the sixth site during the time of prospection, but black fly biting was observed, and rapids were present in the river. Biting was confirmed during prospection, as landing and biting by black flies were observed within less than 5 min of exposing legs at the site. *Simulium* black flies were the main anthropophagic species in the study area.

### *Simulium* larvae susceptibility

Following prospection in the rivers, we had enough larvae to conduct triplicate tests for all test concentrations, but for the controls, we conducted double tests. The sensitivity test results are summarized in Table 1.

**Table 1:** Larvae sensitivity to different temephos concentrations

Temephos concentration (mg/l)	Number of larvae tested	Percent (%) mortality (no. of larvae deaths)
0.1	120	100 (120)
0.05	120	100 (120)
0.025	120	100 (120)
0.01	120	92.5 (111)
0.005	120	70 (84)
0.0025	120	21.67 (26)
0.001	120	12.5 (15)
0 (control)	80	3.8 (3)

Results of susceptibility testing of the larvae revealed that as the concentration of temephos decreased (from 0.1 to 0.001 mg/l), the mortality of *Simulium* larvae decreased proportionally. Mortality of 100% was achieved at concentrations between 0.1 and 0.025 mg/l. The half-maximal effective concentration (EC_50_) for this experiment was 0.0032 mg/l.

**Fig. 3:**
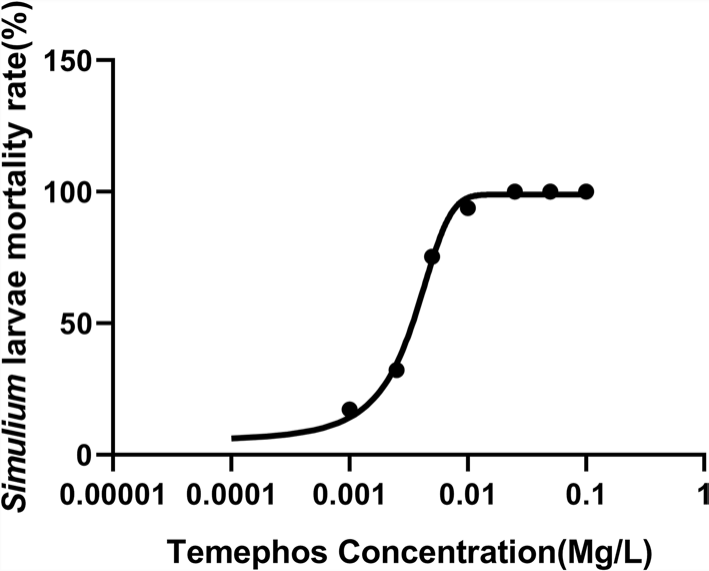
Dosage–mortality response of *Simulium* larvae to temephos

The dosage–mortality regression curve was plotted for susceptibility testing (Fig. 3). The control mortality was set to a concentration of 0.0001 mg/l, as taking a logarithm of 0 is indeterminate. This shows that the concentration of temephos required to kill 50% of larvae (EC_50_) was 0.0032 mg/l. The Hill slope (steepness of the curve) was not constrained and included the control mortality.

### Entomological indicators at baseline

Baseline entomological indices were collected for two consecutive months (June and July 2021).

## Biting density at baseline

**Fig. 4:**
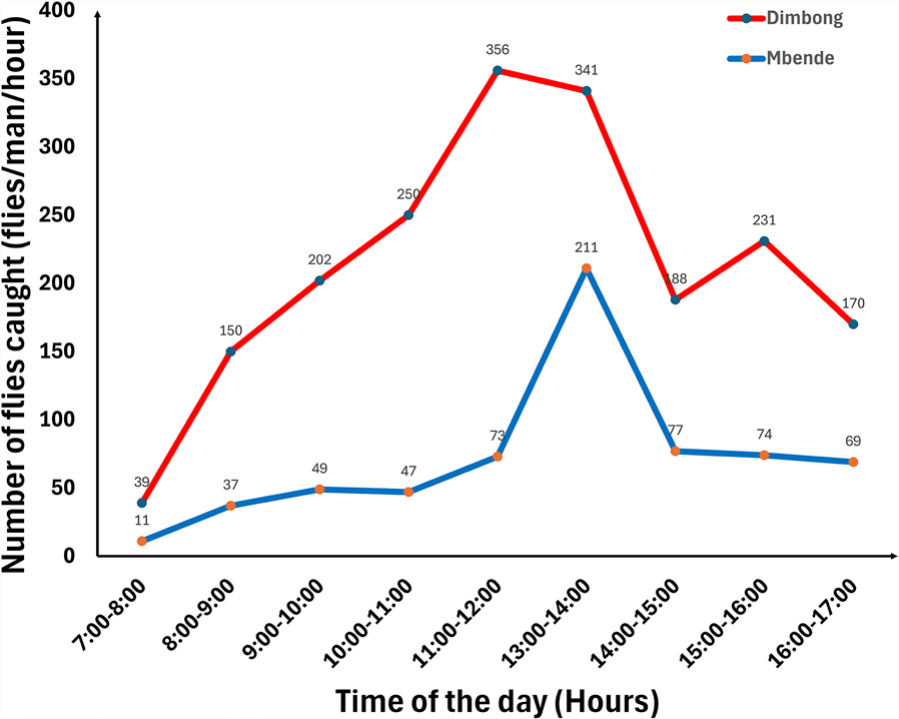
Average hourly biting rates at collection sites, June 2021

At the collection points (banks of Mbende and Dimbong rivers), biting was higher during the afternoon hours, with peaks between 12 noon and 2 pm. On average, the highest number of flies per hour were caught at Dimbong (356 flies/man/h) between 1 and 2 pm (Fig. 4). Table 2 shows the DBR at the collection sites in June 2021.

**Table 2:** Entomology data showing daily biting rates at Mbende and Dimbong rivers in June 2021

Date	DBR at Mbende River	DBR at Dimbong River
17/6/2021	1076	–
18/6/2021	501	327
19/6/2021	697	691
20/6/2021	413	49
Total	2687	1067
	Average DBR = 672 flies/man/day MBR for June 2021 = 20,153 flies/man/month	Average DBR = 356 flies/man/day MBR for June 2021 = 10,670 flies/man/month

DBR = daily biting rate (flies/man/day), MBR = monthly biting rate (flies/man/month)

During the second phase of baseline entomological investigations (July 2021), flies were caught at all hours of the day, with no hour recording zero. The highest number of bites was recorded between 9 and 11 am. A total of 8990 flies were caught at the two riverbanks. A general DBR of 499 flies/man/day and 833 flies/man/day was determined for Mbende and Dimbong rivers, respectively. Table 3 summarizes the DBRs at the collection sites in July 2021.

**Table 3:** Entomology data showing biting rates at Mbende and Dimbong rivers in July 2021

Date	DBR at Mbende River	DBR at Dimbong River
2/7/2021	417	951
3/7/2021	851	900
4/7/2021	601	527
5/7/2021	704	–
6/7/2021	604	1316
7/7/2021	235	853
8/7/2021	240	453
9/7/2021	338	–
Total	3990	5000
	Average DBR at Mbende = 499 flies/man/day MBR for July 2021 = 15,461 flies/man/month	Average DBR at Dimbong = 833 flies/man/day MBR for July 2021 = 25,833 flies/man/month

DBR = daily biting rate (flies/man/day), MBR = monthly biting rate (flies/man/month)

### Non-target fauna in Mbende River

Based on the fishermen and local population, fish species seen and caught from the Mbende River before treatment with temephos included *Ancharius brevibarbis* (“white masharon”), “yellow masharon”, *Tilapia nilotica*, *Cyprinus barbus* (“banga fish”), *Mormyrus lacerda* (“long mouth”), *Gymnarchus niloticus* (“electric fish”), ***Clarias gariepinus*** (“mud fish”), *Clarias mossambicus* (“dog fish”), *Barbus intermedius* (“sadine fish”), *Salmo trutta* (“white fish”), and crayfish. Before application of temephos, the fishermen presented us with 92 fish (white masharon = 9, snake fish = 3, dog fish = 1, banga fish = 17, yellow masharon = 17, white fish = 8, tilapia = 30, sadine fish = 4, mud fish = 1, mbem = 1, long mouth = 1) of different sizes. After the intervention, they presented 103 freshly caught fish (white masharon = 11, snake fish = 2, dog fish = 3, banga fish = 14, yellow masharon = 23, white fish = 9, tilapia = 28, sadine fish = 7, mud fish = 2, long mouth = 2, electric fish = 1) of similar species and sizes.

Other common species of vertebrates observed included giant frogs and crocodiles, in addition to invertebrates. Invertebrate aquatic fauna observed were crabs and larvae of other insects seen during larval prospection, and pictures were taken. Some of these insects were observed on the same substrate as the *Simulium* larvae (Fig. S3). There was no change in the quantity or diversity of non-target aquatic fauna post-treatment.

### Impact of ground larviciding

The impact of ground larviciding was reflected in the decrease in larval density and subsequent disappearance from the substrates, and the decrease in biting rates of adult black flies during the 10-week treatment period.

### Trend in larval density before and during ground larviciding

Before the start of ground larviciding, larvae were present at the main identified breeding sites on natural substrates and traps. In the case of larval density, substrates were not calibrated, but an average of about 50 larvae were found per substrate measuring 10 cm in length. After a week of treatment, fewer than 10 larvae could be seen on substrates, and by the third week of treatment, no larvae could be seen at the sites.

### Trend in biting density

**Fig. 5:**
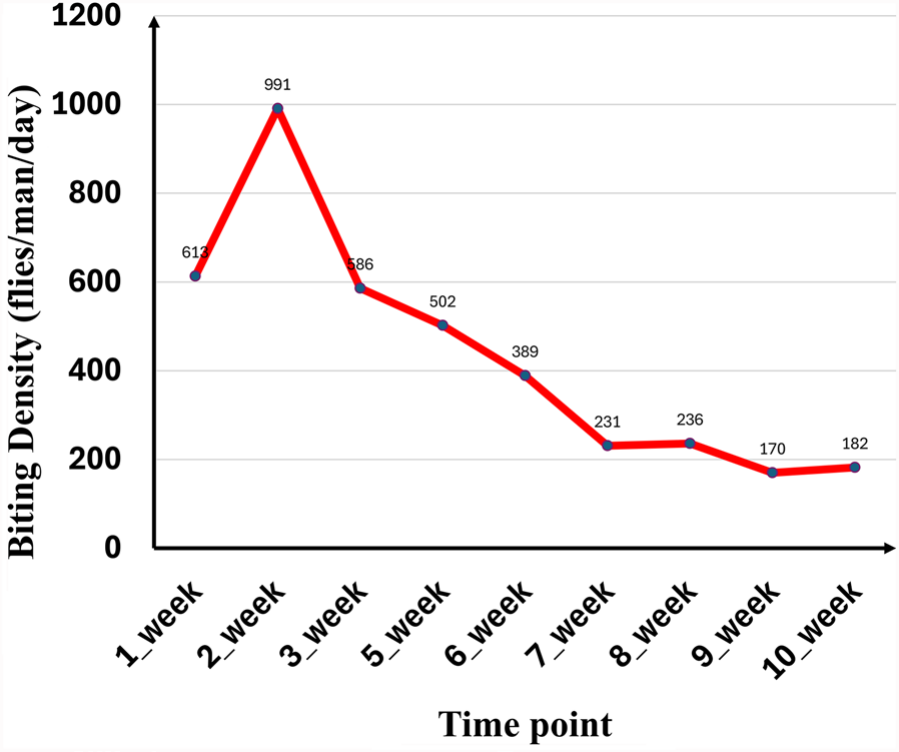
Trend in black fly biting density during the 10 weeks of ground larviciding

The hourly and daily biting rates of adult black flies collected at different time points are shown in Table 4, while the trend in biting density is illustrated in Fig. 5.

**Table 4:** Hourly and daily biting rate at different time points during the 10-week larviciding period

	Time									Daily biting rate (flies/man/day)
Day	7–8 am	8–9 am	9–10 am	10–11 am	11–12 noon	1–2 pm	2–3 pm	3–5 pm	4–5 pm	
Day 1 to first Tm (13/11/2021)	239	42	90	92	0 (rainfall)	28	33	49	40	613
Day 1 to Tm Wk 2	174	189	74	63	135	48	70	66	172	991
Day 1 to Tm Wk 3 (25/11/2021)	45	35	83	109	43	64	39	48	120	586
Day 1 to Tm Wk 5 (7/12/2021)	95	66	20	56	61	43	65	37	59	502
Day 1 to Tm Wk 6 (13/12/2021)	34	67	66	33	32	47	43	55	12	389
Day 1 to Tm Wk 7 (19/12/2021)	26	35	33	20	17	27	09	43	21	231
Day 1 to Tm Wk 8 (25/12/2021)	20	46	33	10	18	21	47	28	16	236
Day 1 to Tm Wk 9 (31/12/2021)	25	31	17	16	11	19	23	15	13	170
Day 1 to Tm Wk 10 (06/01/2021)	27	23	19	21	15	22	17	23	15	182

Tm = treatment; Wk = week

The data collected during this treatment period indicated a very slow but steady reduction in the biting density at the collection point (near the bridge) from week 1 through week 3 to 1 day before week 10 treatment. The highest biting density of 991 flies/man/day was observed on the day before the second treatment, and the lowest of 170 flies/man/day was observed on the eve of the ninth treatment. Overall, a reduction in biting rate of 82.8% was observed following ground larviciding with temephos. The reduction in biting density at the different time points during the 10-week treatment period was statistically significant (*χ*^2^ = 1351.5, *df* = 8, *P* < 0.001).

## Discussion

The study revealed that *Simulium* larvae in the study area were susceptible to temephos (Abate 500 EC [emulsifiable concentrate]) even at concentrations as low as 0.025 mg/l. The susceptibility pattern observed in this study closely matched those reported in the Meme River basin of Cameroon [28] and the Tukuyu onchocerciasis focus in South-West Tanzania [24]. These findings suggest that *Simulium* larvae in forest transmission zones are probably susceptible to temephos. The lethal concentrations of *Simulium* larvae in this study fell within the standard diagnostic dose of ≤ 0.4 mg/l for susceptible *Simulium damnosum* sensu lato (s.l.) populations. This also shows the rapid activity of temephos, which has been shown to be highly efficacious against *S. damnosum* (s.l.) larvae at concentrations giving 0.05 mg/l at high river discharges (> 25 m^3^/s) and at 0.1 mg/l at low discharge (when insecticide carry is reduced). Hence, temephos insecticide could be an effective tool for ground larviciding and accelerating the elimination of onchocerciasis in the Nkam-Wouri River drainage.

We also observed that larvae of other organisms collected with those of *Simulium* were not adversely affected by temephos, partly confirming its safety for river ecosystems. This finding supports the use of temephos for larviciding as an alternative and complementary measure to the CDTi for onchocerciasis control in the study area, which is similar to the approach used on Bioko Island [11]. The dominant non-target, large aquatic fauna in the study river consists of different species of fish, crayfish, and crabs.

Despite the very low biting observed in the mornings, especially when there was a heavy rainfall the previous night, the biting pattern analysis revealed *Simulium* activity at all hours of the day, with no zero-hour recordings in either collection site pre-intervention. Peak biting rates were recorded between 9 am and 2 pm, and this could be because during these hours, the weather is neither too hot nor too cold and thus promotes the activity of the flies.

The reduction in larval density after the first week of river treatment and subsequent non-visualization of black fly larvae on substrates (traps or natural environment) at the breeding sites from the third to the last week of treatment signifies the effectiveness of temephos insecticide in killing and interrupting *Simulium* larval development in the study river. This outcome is similar to what was obtained with biweekly treatment of streams in Lavaderos Valley in Guatemala to eliminate *Simulium ochraceum* larvae within 3 months [29]. However, this treatment effect lasted for only 10 weeks, and breeding sites located many kilometres downstream may not have been adversely affected. Hence, the impact of this intervention could be rapidly reversed in just a few weeks from the last treatment date.

The gradual reduction in the biting densities despite the absence of identifiable larvae in Mbende River from the third to the 10th week was likely due to fly migration from untreated breeding sites both upstream and downstream, and particularly from the nearby untreated Mabombe and Dimbong rivers within the transmission zone. Mabombe River, located just 6 km from our intervention point, maintained identifiable larvae on natural substrates throughout the treatment period. Therefore, the slow reduction in biting rates could be due to flies migrating from the Mabombe River to Solle.

Our findings on the reduction of biting density by 82.8% are very similar to the reported 83.8% reduction in the biting rate of *S. ochraceum* following 3 months of river treatment in Guatemala [29]. On the other hand, our reduction in biting rate was less than that obtained in the Bioko Island study [11], where complete elimination of *Simulium yahense* was achieved. This difference can be attributed to our treatment of only a single river tributary of the Nkam-Wouri for 10 consecutive weeks (2.5 months), whereas on Bioko Island, all the flowing water courses were targeted using helicopters in the hard-to-access areas and complemented with ground-based application of temephos consecutively for 5 months (January to May 2005). To achieve comparable results to the Bioko Island study in Equatorial Guinea, there is a clear need for enlarged ground larviciding, targeting all the river courses within the Nkam-Wouri River drainage. Decreasing vector biting rates below the threshold for endemic transmission will also safeguard elimination once achieved, helping to minimize resurgence or reintroduction of infection.

Anecdotally, during both pre- and post-community and stakeholder engagement and advocacy meetings, community members and local leaders consistently articulated unequivocal support for the study. They highlighted a favourable disposition toward its objectives and a perceived alignment with local health priorities. Their endorsement was evident through active participation, verbal affirmations, and commitments to facilitate the research process. Traditional and local authorities openly acknowledged the study’s relevance, while community representatives pledged their collaboration to ensure smooth implementation. Additionally, post-engagement reflections highlighted sustained willingness among stakeholders to support and advocate for the study’s objectives within their communities. This strong community backing suggests that endemic populations, when well informed about onchocerciasis transmission and pathology, are more likely to support control strategies aimed at accelerating disease elimination. While these findings are interpretative within the study context, they could be extrapolated to areas with similar geo-ecological, socio-demographic, cultural, and disease burdens, where informed community engagement is critical to the success of onchocerciasis elimination efforts.

The high level of acceptance and support could be attributed to the excellent approach used by social scientists in the research team for community engagement. The approach involved individuals and stakeholders from all levels in the community, administration, and health system, while providing a clear and precise picture of the intervention strategy and involving all stakeholders throughout the various phases of the implementation. This aligns with WHO policy, which encourages community-directed methods of vector control that are consistent with the WHO policy on community participation in NTD programmes [30] and help to strengthen the three pillars of the WHO roadmap on NTDs (accelerating programmatic action, intensifying cross-cutting approaches, and facilitating country ownership [5].

While interacting with the population of Solle in a non-systematic manner after the intervention, community members reported experiencing an unusual reduction in black fly presence and biting rates for the first time in recent weeks. They expressed a feeling of excitement about the intervention results.

Through their community leader, the main plea made by the people was to know whether the research team could continue with the treatment of all other rivers within and around the Nkam-Wouri River drainage, to eliminate the nuisance caused by these black fly bites, as well as the burden of onchocerciasis. This community reaction aligns with the findings by Siewe et al. in the Ntui Health District in Cameroon, who reported the local population’s willingness to participate in long-term black fly control to curb or stop the biting nuisance [31]. The request made by the community leader of Solle following this pilot intervention is what we hope should be done on all rivers flowing in the entire Nkam-Wouri River drainage to complement CDTi, hence accelerating onchocerciasis elimination in the area. The anecdotal observations from Solle’s population highlight the need to include a standard qualitative study design during ground larviciding interventions to provide clear explanations about the observed reactions of the people.

This study revealed the susceptibility of *Simulium* larvae to temephos insecticide, resulting in decreased biting density of black flies. The findings demonstrate the feasibility of implementing ground larviciding in Cameroon’s Nkam-Wouri River drainage within the forested ecological zone. However, it was limited in that substrates were not calibrated to better quantify the larval density. Also, the study failed to collect quantitative pre- and post-control data for all non-target fauna for environmental impact assessment. A standard scientific methodology to understand the social impact of the intervention on the people was absent.

## Conclusions

The study demonstrated that *Simulium* larvae in the study area were highly susceptible to temephos insecticide, with complete clearance of identified larvae from both traps and natural substrates following treatment. Ground larviciding significantly reduced *Simulium* biting density, providing temporary relief from vector biting distress in the affected community and potentially decreasing the transmission rate of onchocerciasis, contributing to a reduction in disease burden. These findings suggest that targeted larval control using temephos could be a valuable component in integrated approaches to combat river blindness, particularly in forest endemic zones, warranting further exploration in similar endemic regions.

## Supplementary Information

Supplementary material 1: Fig. S1. Setting of traps for *Simulium* larvae breeding. Palm fronts carried by members of the research team, substrates placed in rapids points in the river to serve as traps for *Simulium* breeding.

Supplementary material 2: Fig. S2. *Simulium* larvae in bowls, placed in a locally made wooden box having a transparent glass, containing ice packs and a thermometer to control incubation temperatureduring sensitivity testing. Side viewand front viewof pictures taken during the incubation period.

Supplementary material 3: Fig. S3. Non-target invertebrate aquatic fauna. On a leafand from the tree branch substrate with *Simulium* larvae.

## Data Availability

All data analysed during this study are included within the paper and the supplementary files.

## Abbreviations

APOC: African Programme for Onchocerciasis Control
CDTi: Community-directed treatment with ivermectin
DBR: Daily biting rate
MBR: Monthly biting rate
MDA: Mass drug administration
OCP: Onchocerciasis Control Programme
TNT: Test and treat
