# Supplementary figures and images for: Simulium larvae susceptibility to temephos and the effect of 10 weeks of treatment of the Mbende tributary in the Nkam-Wouri River drainage of Cameroon on larval density and adult fly biting rates
Source: Parasit Vectors. 2025 Jul 1;18:242. doi: 10.1186/s13071-025-06837-5 (PMC12210698; doi:10.1186/s13071-025-06837-5)

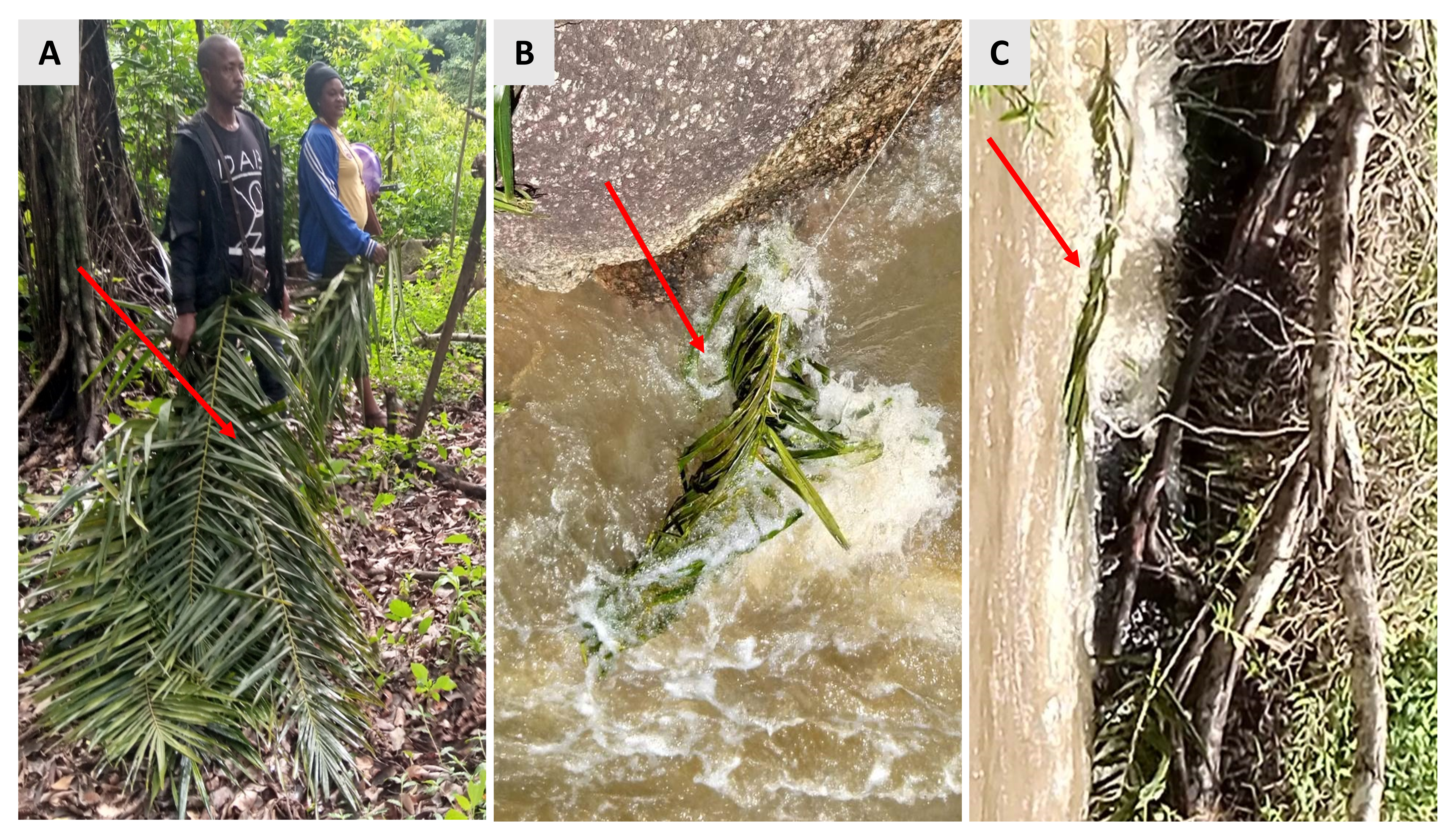

Supplement: Supplementary file 1 — Supplementary material 1: Fig. S1. Setting of traps for Simulium larvae breeding. Palm fronts carried by members of the research team, substrates placed in rapids points in the river to serve as traps for Simulium breeding. [file 13071_2025_6837_MOESM1_ESM.tif]

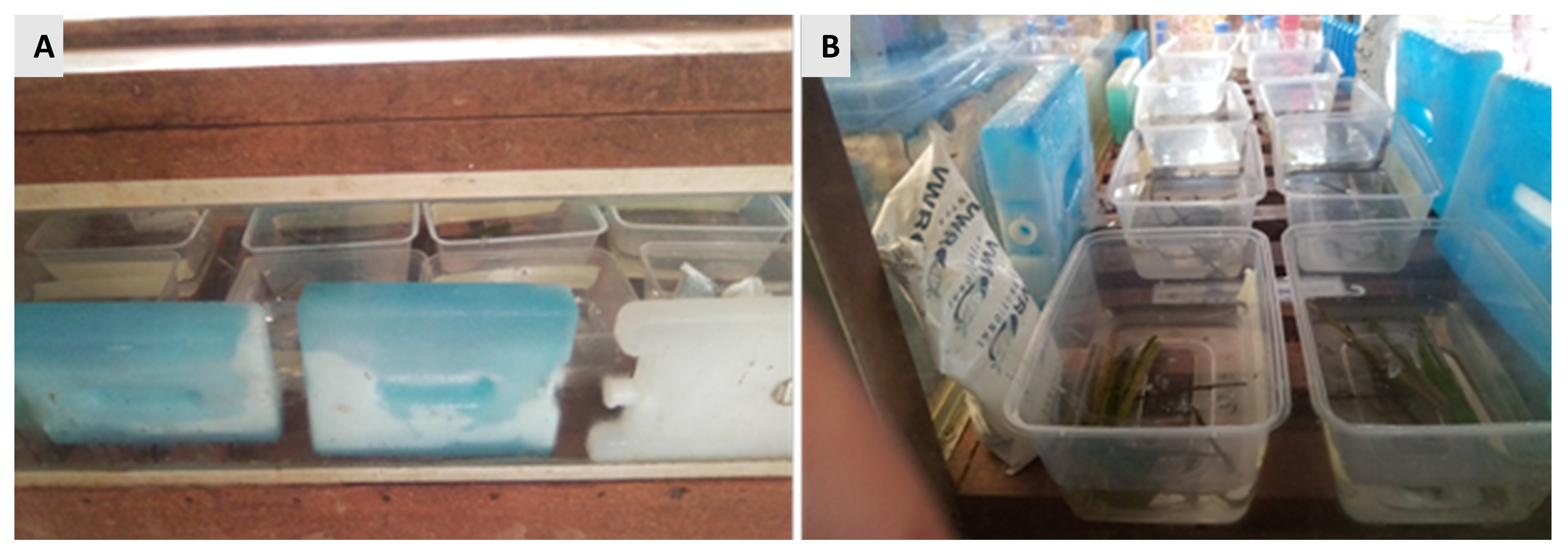

Supplement: Supplementary file 2 — Supplementary material 2: Fig. S2. Simulium larvae in bowls, placed in a locally made wooden box having a transparent glass, containing ice packs and a thermometer to control incubation temperatureduring sensitivity testing. Side viewand front viewof pictures taken during the incubation period. [file 13071_2025_6837_MOESM2_ESM.tif]

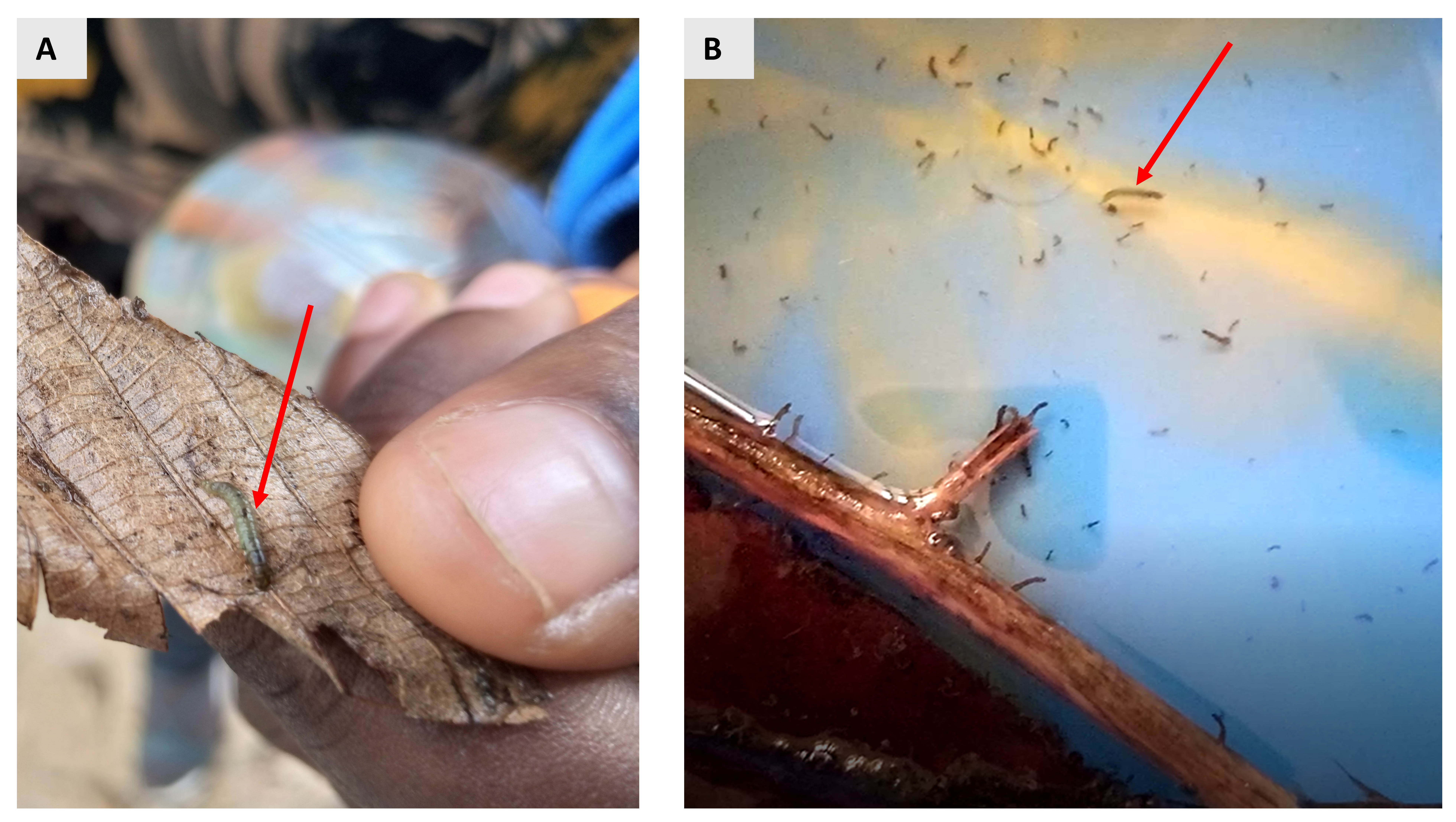

Supplement: Supplementary file 3 — Supplementary material 3: Fig. S3. Non-target invertebrate aquatic fauna. On a leafand from the tree branch substrate with Simulium larvae. [file 13071_2025_6837_MOESM3_ESM.tif]
